# Reliability and validity of sprint performance using the Alex7 motorized device

**DOI:** 10.3389/fspor.2024.1412861

**Published:** 2024-07-25

**Authors:** Austra Skujytė, Inga Lukonaitienė, Jūratė Stanislovaitienė, Viktoras Šilinskas, Kristina Bradauskienė, Gediminas Mamkus, Sigitas Kamandulis

**Affiliations:** ^1^ Institute of Sports Science and Innovation, Lithuanian Sports University, Kaunas, Lithuania; ^2^ Sports Coaching Department, Lithuanian Sports University, Kaunas, Lithuania

**Keywords:** test-retest, kinematic parameters, assisted sprinting, resisted sprinting, youth, football

## Abstract

**Introduction:**

Advancements in technology have recently made it possible to implement effective training solutions across different environmental conditions. This study evaluated the reliability and validity of measures obtained from the innovative motorized device, Alex7 (Inosportas, Lithuania), and differences in speed and kinematic characteristics between resisted and assisted sprinting in young football players.

**Methods:**

Twenty-seven male athletes (mean age: 16.5 ± 0.8 years; height: 179.5 ± 6.9 cm; body weight: 67.7 ± 8.3 kg) each performed 30-m sprints twice under three different conditions: regular, resisted, and assisted sprinting. The Alex7 device provided the assistance and resistance during sprints. Results were compared with those from Witty timing gates. Ground contact time, flight time, stride length, and pace were measured using the OptoJump system. Reliability was assessed using two-way mixed intraclass correlation coefficients (ICCs) for single measures, the standard error of the mean (SEM), and the coefficient of variation (CV). Pearson's correlation coefficient determined the associations between Alex7 and Witty timing systems. Criterion-referenced validity was based on the mean difference and CV. Systematic bias was determined by limits of agreement using Bland–Altman analysis.

**Results:**

Running times obtained using the Alex7 equipment exhibited good to excellent test-retest reliability between sessions (ICC, 0.83–0.94) and good to excellent correlation (Pearson's *r* = 0.88–0.98) between the Alex7 and Witty systems in both assisted and resisted running conditions. However, the Alex7 device consistently produced longer running times than the Witty device (up to 0.16 s difference, *p* < 0.001). The different running conditions produced substantial variations in kinematic variables, such as stride length, ground contact time, and running speed (*p* < 0.001 for all), but the effects on flight time and running pace were smaller.

**Discussion:**

The Alex7 device shows high reliability for creating resisted and assisted running conditions for young football players. However, it tends to overestimate running time, necessitating caution when assessing the time parameters.

## 1 Introduction

To enhance sprint performance, methods range from high-magnitude resisted overload at low velocity to unloaded assisted sprinting using elastic cords, bungees, or pulleys (1–4). These two opposite training modalities induce specific neural and muscular adaptations (5), which can be measured and expressed in the force–velocity relationship (6, 7). The maximum performance of the neuromuscular system is determined by the intercepts in this relationship, although controlling resisted and assisted testing and training is difficult and requires skill. This becomes more challenging in team sports such as football because of the training complexity and limited time available to develop both speed and power.

Recent technological advances have enabled practical solutions for training under varying environmental conditions (8, 9). Several robotic devices are used to provide resisted or assisted motorized towing for sprint assessment and training (DynaSpeed, 1080 Sprint). These motorized devices allow for greater accuracy in setting the resisted and assisted load magnitude. A new device, Alex7 (Inosportas, Lithuania), has been developed recently. Similar to other systems, the Alex7 provides resistance (up to 30 kg) with a cable to prevent full speed running or assists at a speed (up to 14 m/s) that is faster than the maximum achieved during regular sprinting. It also offers assessments of velocity and displacement for real-world data collection, facilitating individualized sprint training. High performers can especially benefit from actual training data (10), but this information is useful only if the new equipment provides accurate and consistent results.

Given the novelty of the Alex7, this study primarily aimed to establish its reliability and validity. The secondary aim was to compare the differences in speed and kinematic characteristics between resisted and assisted sprints to identify individual variability between young athletes in a football squad. It was expected that the Alex7 would prove reliable and useful for measuring and developing young football players’ sprint capacity.

## 2 Materials and methods

### 2.1 Participants

Twenty-seven young male football players competing in top-tier U16 and U18 leagues (age, 16.5 ± 0.8 years; stature, 179.5 ± 6.9 cm; body weight, 67.7 ± 8.3 kg) were recruited. *a priori* power analysis (G*Power version 3.1.9.7, University of Düsseldorf, Germany) indicated that a minimum sample size of 23 participants was required, with an alpha level (*α*) of 0.05 and a statistical power of 0.80. This calculation was based on a pilot study in which 14 young basketball players performed a 30-meter sprint twice, resulting in an effect size of 0.82 for the time difference between trials. The participants had no recent injuries or medical conditions that could interfere with maximal exertion in each sprint trial. All participants regularly performed sprinting during their training sessions, but none had prior experience in completing motorized resisted or assisted sprinting. After verbal and written explanation of the experimental design and its potential risks and benefits, written informed consent was obtained from all players and their respective parents/guardians. The study adhered to the high ethical guidelines in sports sciences (11). Ethical approval was received by the local Institutional Review Board (code: MNL-TRS (M)-2023-606).

### 2.2 Design

**Figure 1:**
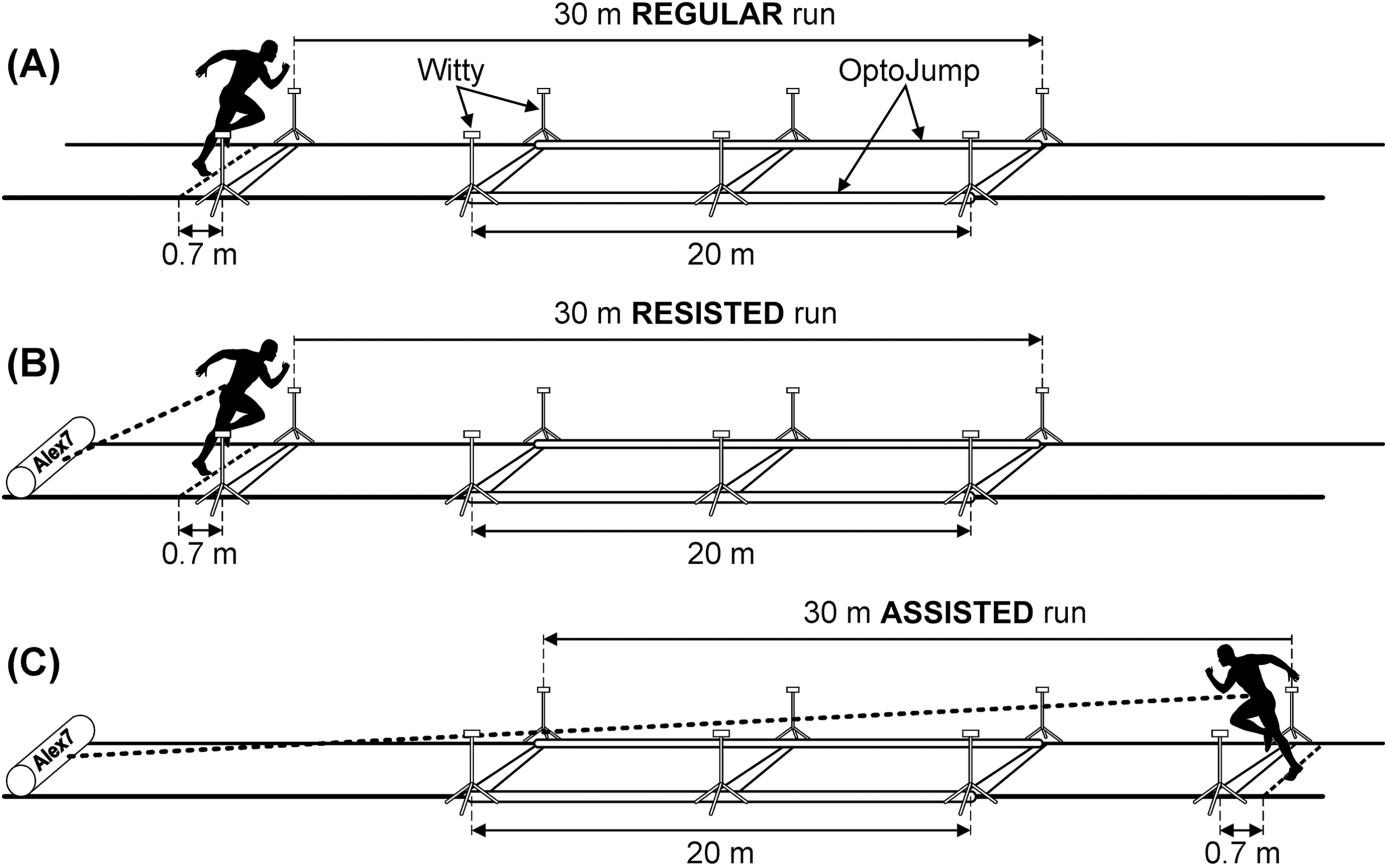
Schematic diagram of the experimental testing setup for regular (**A**), resisted (**B**), and assisted (**C**) sprint testing. Three different devices (OptoJump, Witty, and Alex7) were used during each trial.

The kinematic running parameters and their changes during a 30-m sprint were measured using three types of equipment (Witty, OptoJump, and Alex7) under the following conditions: regular (no resistance or assistance), with resistance, and with assistance created by the motorized Alex7 device (Figure 1). The testing sessions were conducted in a repeated manner on a weekly basis. The initial week was designated for regular running and familiarization with the resisted and assisted sprinting conditions. In the following 2 weeks, the testing was conducted identically and incorporated both resisted and assisted sprinting, and the results were used to assess the reliability and validity of the Alex7 device. All testing sessions for each participant occurred at about the same time of day on the same weekday. The primary outcome variables were sprint time and velocity, ground contact time, flight time, stride length, and pace.

### 2.3 Procedures

All testing occurred on a rubberized indoor track with a 60-m straightway. During the first testing day, height (to the nearest 0.1 cm, Martin-type anthropometer, GPM instrument, Siber-Hegner, Switzerland) and body mass (to the nearest 0.1 kg, TBF-300 Body Composition Analyzer, Tanita, Philpots Close, UK) were recorded. Before each testing session, a standardized 20-min warm-up that included jogging, dynamic stretches, specific running exercises, and submaximal sprints was performed (8, 12).

Running times for the entire 30 m, with 10-m and 20-m splits, were recorded for each condition (regular, resisted, and assisted) using Witty photocells (Microgate, Bolzano, Italy). The participant began each sprint trial with his lead foot placed 70 cm behind the first photocell to prevent inadvertent triggering of timing in the start position (13, 14). Two sprint trials were conducted with a minimum of 6 min of passive recovery between each trial in each condition (8). The fastest sprint time was used in the analysis of each condition and each testing session (13).

During each sprint trial, the kinematic parameters of velocity, ground contact time, flight time, stride length, and pace were measured over 10–30 m using the OptoJump modular system (Microgate, Bolzano, Italy), an optical data acquisition system comprising two parallel bars containing light-emitting diodes that detect any interruption in communication between the bars (Figure 1). The kinematic parameters were computed based on the data obtained from the four fastest steps performed by each participant. Calculations of kinematic parameters during running measured using the OptoJump have been reported to have high validity [intraclass correlation coefficient (ICC), 0.96–0.99; mean bias, 0.4%–2.7%] (15).

During the resisted and assisted trials, the participants sprinted while wearing a waist harness connected to the Alex7 composite fibre cable (High Tenacity Polyester and Dyneema®12 strand braid), which was wrapped around a 90-mm diameter spool and extendable up to 31 m in length. The cable load was controlled using a servomotor (Mitsubishi HG-SR152) and the accompanying programmable controller. During the resisted run, the participant ran with the resistance attached to the cable set at 10% of body weight. In the assisted trial, the participant was pulled with an assisted load of 6 kg. This load was selected based on a previous investigation that reported a substantial increase in maximal velocity using a 7-kg load in sprinters (8) but less of an effect using a 6-kg load in football players lacking experience of being pulled with a motorized device.

The Alex7 device applied resistance or assistance, and the timing was initiated immediately at the start of the sprint, that is, 0.7 m before the athlete reached the first photocell of the Witty timing gate. The time interval corresponding to the initial 0.7 m was extracted from the Alex7 device and was used to measure the 30-m sprint time and to calculate the 10-m and 20-m split times in each experimental condition. Per the manufacturer's recommendations, all trials using the Alex7 were completed in the Isotonic mode. The controller measures the distance travelled by the sprinter and was programmed to terminate the assisted load at the finish line to allow the participant to decelerate safely after completing the maximum-velocity portion of the sprint.

### 2.4 Statistical analysis

Mean and standard deviation (SD) were presented for all sprint times and kinematic parameters. The Shapiro–Wilk test confirmed normal data distribution. Relative reliability was assessed using two-way mixed ICCs for single measures, interpreted as moderate (0.50–0.74), good (0.75–0.89), and excellent (≥0.90) (16). Absolute reliability was assessed with the standard error of the mean (SEM), calculated as SD/√2, and coefficient of variation (CV), computed as SD/mean × 100%. Pearson's correlation coefficient (r) determined the associations between Alex7 and Witty timing systems, interpreted as large (0.50–0.69), very large (0.70–0.89), nearly perfect (0.90–0.99), and perfect (1.00) (17). For criterion-referenced validity, mean difference (tdiff) and CV were calculated, with paired sample t-tests testing the significance of mean differences. Cohen's d was used for effect sizes, interpreted as large (≥0.80), medium (0.50–0.79), small (0.20–0.49), or trivial (<0.20) (18). Bland-Altman analyses illustrated agreement between repeated measures for Alex7 and between Alex7 and Witty timing systems (19). The 95% CI of the mean difference determined systematic bias. All statistical analyses were performed using IBM SPSS Statistics (version 28.0, Armonk, NY, USA), with significance set at *α* = 0.05.

## 3 Results

**Figure 2:**
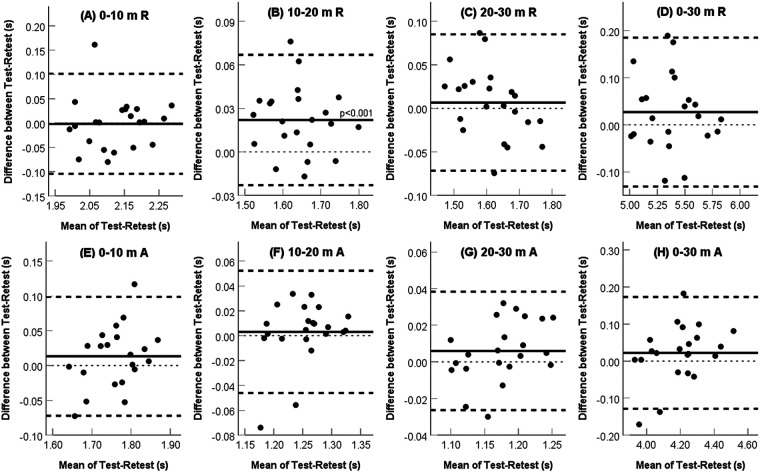
Bland-Altman analysis of test-retest sprint times derived from Alex7. The plot differences were obtained by subtracting the retest value from the test value. Thick black solid line – mean difference between the two tests; thick black dashed lines – 95% limits of agreement; thin black dashed line – zero value.

The descriptive statistics for the between-session comparison during the resisted and assisted running split times using the Alex7 are presented in Table 1. Running times measured with the Alex7 demonstrated good to excellent test-retest reliability (ICC, 0.83–0.94) in both assisted and resisted conditions. The reliability of the split time tended to be lower in the first part of the course (ICCs of 0.83–0.86 for 0–10 m) in both assisted and resisted conditions. The CV was 0.80%–1.43%, and the SEM was 0.01–0.05 s for the time intervals independent of the running condition. In contrast to the other variables, the split time for 10–20 m in the resisted condition improved significantly and consistently from the first testing session to the retest (*p* < 0.001). Notably, this improvement was accompanied by a similar level of reliability as that for the other markers assessed. In addition, Bland-Altman analyses showed no significant systematic bias between repeated measurements except for the 10–20 m split time in the resisted condition (*p* < 0.001, Figure 2). The narrow range of limits of agreement on the Bland-Altman plot indicates high stability and low variation between test occasions. Repeatability for most measures was within the 95% CI.

**Table 1:** Between-session reliability of the Alex7 outcome time and optoJump kinematic variables.

	Test (mean ± SD)	Retest (mean ± SD)	Mean difference ± SD	*p* =	ICC	ICC (95% CI)	CV (%)	SEM (s)
Running splits								
R 0–10 m	2.13 ± 0.09	2.13 ± 0.09	−0.002 ± 0.053	0.890	0.83*	[0.64 to 0.93]	1.24	0.03
R 10–20 m	1.65 ± 0.07	1.63 ± 0.07	0.022 ± 0.023	<0.001	0.92*	[0.53 to 0.98]	1.12	0.02
R 20–30 m	1.62 ± 0.08	1.61 ± 0.10	0.007 ± 0.040	0.444	0.90*	[0.77 to 0.96]	1.40	0.02
R 0–30 m	5.39 ± 0.24	5.37 ± 0.25	0.027 ± 0.081	0.131	0.94*	[0.86 to 0.98]	0.85	0.05
A 0–10 m	1.77 ± 0.08	1.76 ± 0.07	0.013 ± 0.044	0.167	0.83*	[0.63 to 0.92]	1.43	0.03
A 10–20 m	1.25 ± 0.05	1.25 ± 0.04	0.003 ± 0.025	0.573	0.86*	[0.70 to 0.94]	0.95	0.01
A 20–30 m	1.18 ± 0.05	1.17 ± 0.04	0.006 ± 0.016	0.105	0.94*	[0.85 to 0.97]	0.80	0.01
A 0–30 m	4.21 ± 0.18	4.18 ± 0.15	0.022 ± 0.077	0.188	0.88*	[0.74 to 0.95]	1.01	0.04
Kinematic variables								
R Contact time (s)	0.15 ± 0.01	0.15 ± 0.01	−0.002 ± 0.005	0.066	0.88*	[0.73 to 0.95]	2.16	0.00
R Flight time (s)	0.09 ± 0.01	0.09 ± 0.01	0.001 ± 0.007	0.596	0.79*	[0.56 to 0.91]	3.93	0.00
R Stride length (m)	3.18 ± 0.14	3.25 ± 0.16	0.069 ± 0.094	0.003	0.74*	[0.31 to 0.90]	1.74	0.06
R Pace (step/s)	4.17 ± 0.20	4.13 ± 0.23	0.044 ± 0.127	0.116	0.82*	[0.61 to 0.92]	1.72	0.07
A Contact time (s)	0.12 ± 0.01	0.12 ± 0.01	0.000 ± 0.006	0.951	0.85*	[0.66 to 0.93]	2.82	0.00
A Flight time (s)	0.11 ± 0.01	0.11 ± 0.01	0.003 ± 0.006	0.079	0.79*	[0.55 to 0.91]	3.41	0.00
A Stride length (m)	4.13 ± 0.18	4.19 ± 0.20	0.059 ± 0.090	0.005	0.85*	[0.57 to 0.94]	1.40	0.06
A Pace (step/s)	4.28 ± 0.23	4.23 ± 0.22	0.044 ± 0.128	0.124	0.83*	[0.63 to 0.93]	1.70	0.07

ICC, interclass correlation coefficient (*p < 0.001); CI, confidence interval; CV, coefficient of variation; SEM, standard error of the mean; R, running with resistance; A, running with assistance.

Stride length differed between the two testing sessions for both the resisted and assisted sprints (*p* < 0.05, Table 1) but not for other kinematic variables. Almost all kinematic variables showed good test–retest reliability (ICC, 0.79–0.88), except for a moderate value for stride length (ICC, 0.74) in the resisted sprint and an excellent value for running speed in the assisted sprint (ICC, 0.94). In general, the ICCs for almost all variables were slightly higher in the assisted than the resisted sprints.

**Figure 3:**
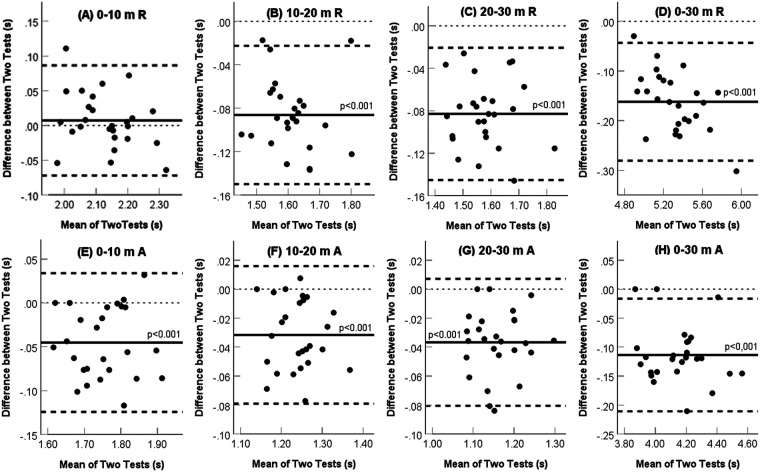
Bland-Altman analysis of sprint time differences between witty and Alex7 equipment. The plot differences were obtained by subtracting the Alex7 test value from the Witty test value. Thick black solid line – mean difference between the two tests; thick black dashed lines – 95% limits of agreement; thin black dashed line – zero value.

All split times in the resisted sprints and all split times except the first (0–10 m) in the assisted sprint had excellent Pearson's correlation coefficient values (r = 0.90–0.98, *p* < 0.001; Table 2) between the Alex7 and Witty equipment. The first 10-m assisted running time had a slightly lower but still a good value (r = 0.88, *p* < 0.001). However, the Alex7 consistently recorded longer running times compared to the Witty device (up to a 0.16 s difference, CV, 1.00%–3.79%, *p* < 0.001). Furthermore, there was a negative bias between both pieces of equipment for both conditions (*p* < 0.001, Figure 3), except for the 0–10 m split time in the resisted run. Still, sprint time remains within a narrow range of limits of agreement on the Bland-Altman plot, and repeatability for most measures was within the 95% CI. These data suggest that the Alex7 system is consistent but is not as accurate for measuring sprint time. The time differences between the two types of equipment were slightly smaller for assisted (range, 0.03–0.11 s) than for resisted (range, −0.01 to 0.16 s) sprinting.

**Table 2:** Within-session criterion-referenced validity for the Alex7.

Conditions	Distance	Sprint time (Mean ± SD)		*p*	ES	Criterion-referenced validity		
		Witty (s)	Alex7 (s)		Cohen's d	t(diff) (s)	CV (%)	Cor. (r)
Resisted	0–10 m	2.13 ± 0.09	2.13 ± 0.10	0.373	0.178	–0.01	1.00	0.92*
	10–20 m	1.57 ± 0.08	1.65 ± 0.09	<0.001	−2.655	0.09	3.79	0.93*
	20–30 m	1.54 ± 0.09	1.62 ± 0.10	<0.001	−2.609	0.08	3.73	0.94*
	0–30 m	5.23 ± 0.25	5.40 ± 0.28	<0.001	−2.678	0.16	2.14	0.98*
Assisted	0–10 m	1.73 ± 0.08	1.78 ± 0.08	<0.001	−1.122	0.05	1.93	0.88*
	10–20 m	1.22 ± 0.05	1.26 ± 0.06	<0.001	−1.307	0.03	1.84	0.90*
	20–30 m	1.14 ± 0.06	1.18 ± 0.06	<0.001	−1.643	0.04	2.25	0.93*
	0–30 m	4.09 ± 0.18	4.21 ± 0.19	<0.001	−2.223	0.11	1.93	0.97*

t_(diff)_, time difference between the systems; CV, coefficient of variation; *p*, *p*-value of the paired samples *t*-test for the difference between Witty and Alex7; ES, effect size; Cor., Pearson's correlation coefficient (*p < 0.001).

**Figure 4:**
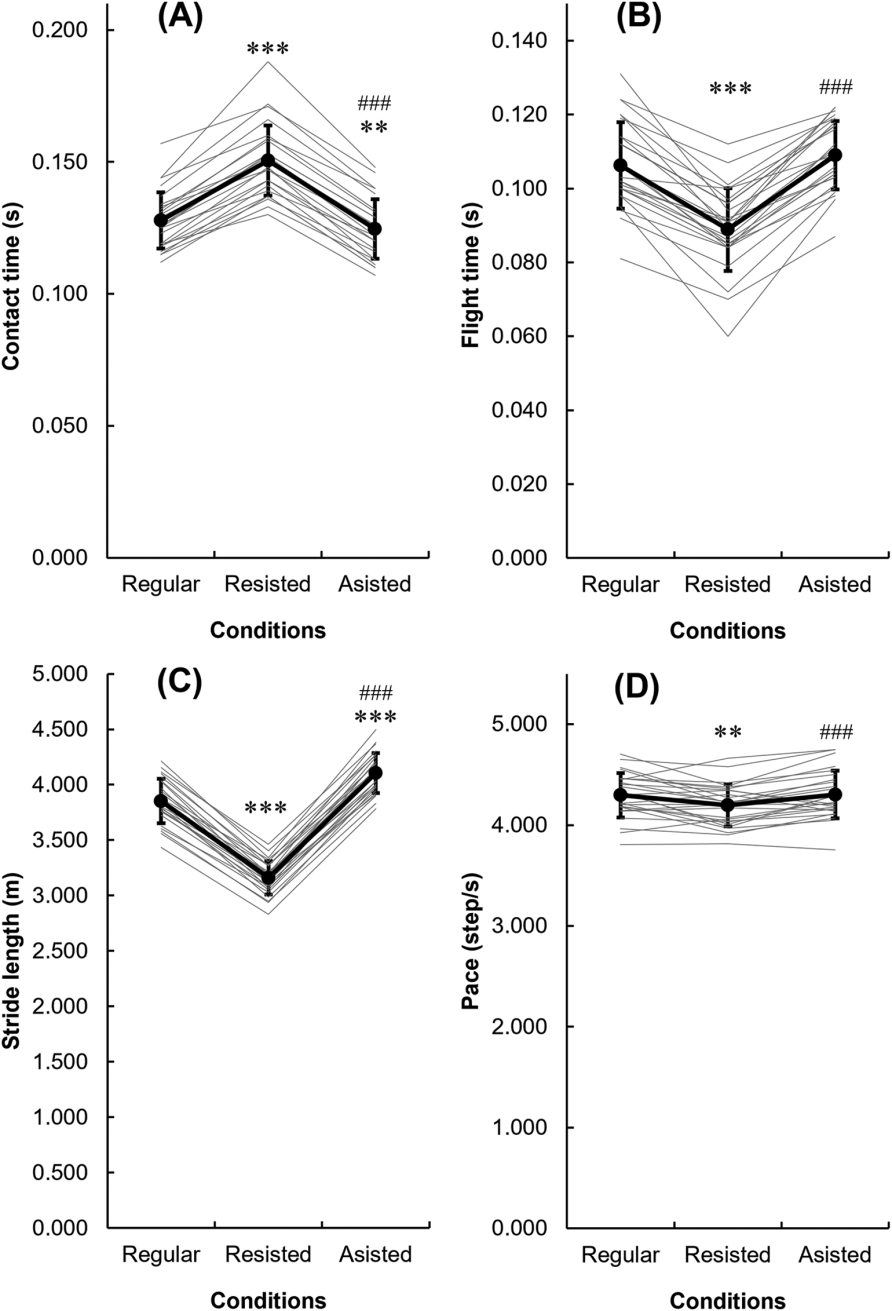
The contact time (**A**), flight time (**B**), stride length (**C**), and pace (**D**) for the regular, resisted, and assisted sprints (mean ± SD and individual data). Significantly different from regular sprints (**p < 0.01, ***p < 0.001). Significantly different from resisted sprints (### *p* < 0.001).

The mean running speed differed between three sprint conditions: 8.17 ± 0.39 m/s in the regular run, 6.59 ± 0.35 m/s in the resisted run (*p* < 0.001), and 8.65 ± 0.44 m/s (*p* < 0.05) in the assisted run. All kinematic variables investigated (Figures 4A–D) differed between the sprint conditions. The largest differences were observed for stride length and ground contact time (*p* < 0.001 for each). The smallest differences were for flight time and running pace. The running flight time and pace did not differ between the regular and assisted runs but differed significantly between the regular and resisted sprints, and between the resisted and assisted sprints (*p* < 0.001 for each). All variables showed a greater absolute difference between the resisted and regular sprints than between the assisted and regular sprints.

## 4 Discussion

The reliability and validity of measurements obtained using the new Alex7 motorized sprint device were investigated to determine whether and how speed and kinematic characteristics differ between assisted and resisted sprints. The primary finding is that the Alex7 device offers reliable measures of sprint performance in both resisted and assisted conditions. However, it is important to note that the Alex7 consistently overestimated running times compared to the Witty device. This overestimation may result from various factors, including differences in measurement methodologies or the specific mechanics of the Alex7 device. Despite this limitation, the Alex7's ability to deliver consistent results highlights its utility for manipulating the load during athlete training. Future research should aim to better understand the causes of the Alex7's running time overestimation and explore methods to mitigate this issue.

In the context of load management, the use of incline, decline, sleds, bungees, and pulleys serves helps to induce the acute effects of overload- or unloaded-assisted stimulation (20), whereas motorized devices such as Alex7 are designed specifically to offer a higher degree of accuracy in load management compared with other devices. The reliability values of the 30-m sprint run time and the split times for each 10 m (ICC, 0.83–0.90) obtained during the resisted running mode with the Alex7 were found to be similar to the within-session reliability values reported by Rakovic et al. (9) for the 1080 Sprint device (ICC, 0.81–0.95). Rakovic et al. (9) showed that the split time for the initial 5 m was the least reliable measurement (ICC, 0.81), indicating less reliability for use with short accelerations. In the present study, this finding was partially replicated using a running distance of 10 m instead of 5 m, yielding an ICC of 0.83. Assisted running was also examined and compared with resisted running, showing a similar trend. That is, the lowest reliability value (ICC, 0.83) was obtained for distances of 10 m, whereas medium reliability values were recorded for the 10-m and 20-m split times and total distance of 30 m (ICC = 0.86 and 0.88, respectively). Notably, the highest reliability value was achieved for the 20–30-m split time (ICC = 0.94), all of which had acceptable reliability when used with the new device for facilitating speed training with assisted running.

The Alex7 can provide both contrasting loads and a high variety in magnitude as well as the opportunity to monitor performance and progress. Compared with the Witty timing gates, which were used as the gold standard in this study, the Alex7 device showed strong associations (r = 0.88) for the 10-m split time in the assisted run, and the other correlations for the assisted runs and all correlation values for the resisted runs were nearly perfect (r = 0.90–0.98). Different results were observed for the 1080 Sprint device in the resisted running mode (9). Nearly perfect validity was found for the 20–30-m split time and the overall 30-m time, but the other correlation values ranged from r = 0.57 to r = 0.83. These differences in validity between the Alex7 and 1080 Sprint devices may reflect differences in the data collection. For example, unstable results were observed for the first 0.7 m due to the participants' inconsistent starting patterns. After being told to prepare for the run, some participants were unable to remain in the static starting position, and their in-place movement started timing before the actual run began. Removing the results of the first 0.7 m eliminated this inaccuracy and produced higher validity values for the first 10 m.

Real-time values from the Alex7 device should be used cautiously because they were significantly longer than those obtained from the Witty timing gates. A consistent discrepancy was observed across all distances, except for the initial split during resistive runs, where no such disparity was noted. This disparity may be attributed to limitations imposed by the Alex7 device in terms of time recording. The controller calculates the distance travelled in meters by multiplying the current number of revolutions (encoder pulses) by circumference. A random error in the calculated length may occur because of differences in the tightness with which the rope was wound on the spool between the assisted and resisted runs and the rope elasticity (although <1%). After completion of the resisted run, the rope was recoiled freely back to its starting position, and during the assisted run, the athlete created a resistance for the servomotor and the rope was wrapped tensely around the spool.

It is important that the new Alex7 device may produce meaningful differences in the kinetics and kinematics parameters between assisted and resisted running conditions. The effects on running speed and kinematics depend on the magnitude of the resistance or assistance. The 6-kg (∼60 N) assistance in the present study caused significant changes in stride length (6.75%), ground contact time (–7.69%), and running speed (6.74%). The running pace and flight time did not differ significantly between the regular and assisted runs. Similar findings have been reported by Cecilia-Gallego et al. (21). In the study by Van Den Tillaar et al. (22), the 5-kg assistance provided by the motorized DynaSpeed device produces a 5.1% increase in speed, but the running pace did not differ between the assisted and unloaded sprints. In another study that used a load of 4.7% of body weight, running speed increased by 6.1%, but the running flight time did not differ significantly (23). In another study, use of a pulling device decreased the contact time by 7.69% (24). The data for the current study suggested that the 6-kg force used for assistance was sufficient to achieve the required effects of overspeed running without interfering with the running technique.

The resistance of 10% of body mass was enough to meaningfully reduce all the running parameters. Compared with the regular sprint, resisted sprinting caused larger changes in running parameters than assisted running. A previous study showed that a resistance of 5% of body mass elicits significant changes in running speed and stride length but that ≥15% of body mass resistance is needed to change stride frequency (25). Given that the motorized device provides greater resistance (by eliminating the inertia) than a sled of the same weight and that 10%–20% of body mass resistance provided by the motorized device can induced greater decreases in speed (13%–28%) compared with a weighted sled with the same weight (7.5%–20%) (26), it was not surprising that the resistance of 10% body weight sufficient enough to significantly decrease (by 2.33%; *p* < 0.01) running pace and running speed (by 19.98%; *p* < 0.001). We note that the participants were non-elite and young (16–17-year-old) football players with no experience in resisted or assisted running. They completed one familiarization session, and this was sufficient to produce high reliability while not altering the running technique. An exception was observed with the split time for 10–20 m in the resisted condition, which improved consistently from the first to the second testing session, indicating a possible need for additional familiarization. Overall, it appears that the Alex7 technology is simple to learn and use in both resisted and assisted training settings, which is important for practitioners.

This study includes several limitations, mainly due to logistical reasons. There is no direct comparison of the Alex7 with well-established systems such as 1080 Sprint or Dynaspeed, which means we lack confirmation that the resistance or assistance is comparable to other similar systems. It would be better to compare timing data with laser systems, which are more accurate than Witty timing gates as they do not rely on cutting light beams with the upper body, making them better suited as gold standards. Finally, only young males were tested, making it unclear whether age, gender, or sport could influence the results. It is reasonable to expect that greater maturity and sprint experience might have positively affected the results.

## 5 Conclusions

The Alex7 device demonstrates high reliability for creating resisted and assisted running conditions for young football players. However, its tendency to overestimate running times necessitates caution when interpreting time parameters. Future research should aim to refine the Alex7's measurement accuracy and explore its applications across various sports, gender and age groups. Additionally, investigating the underlying factors contributing to the observed overestimation of running times could enhance the device's accuracy.

## Data Availability

The original contributions presented in the study are included in the article/Supplementary Material, further inquiries can be directed to the corresponding author.
